# Supplementary material for: Vaccine effectiveness against severe COVID-19 outcomes within the French overseas territories: A cohort study of 2-doses vaccinated individuals matched to unvaccinated ones followed up until September 2021 and based on the National Health Data System
Source: PLoS One. 2022 Sep 9;17(9):e0274309. doi: 10.1371/journal.pone.0274309 (PMC9462750; doi:10.1371/journal.pone.0274309)
Supplement: S1 Table — (DOCX) [file pone.0274309.s001.docx]

**S1 Table.** Vaccine effectiveness against COVID-19-related hospitalisation depending on censoring variable.

| **Model** | **Vaccine exposition** | **Number of subjects** | **Number of event (%)** | **Median follow-up [interquartile range]** | **Crude HR** | **Adjusted HR** | **% risk reduction** |
| --- | --- | --- | --- | --- | --- | --- | --- |
|  |  |  |  |  | **(95% CI)** | **(95% CI)** |  |
| 0 | no | 276778 | 1465 (0.53%) | 77 [42 - 111] | Reference | Reference | - |
|  | yes | 276778 | 96 (0.03%) | 77 [42 - 111] | 0.06 (0.05 - 0.08) | 0.06 (0.05 - 0.07) | 94% (93% ; 95%) |
| 1 | no | 276778 | 1459 (0.53%) | 77 [42 - 111] | Reference | Reference | - |
|  | yes | 276778 | 95 (0.03%) | 77 [42 - 111] | 0.06 (0.05 - 0.08) | 0.06 (0.05 - 0.07) | 94% (93% ; 95%) |
| 2 | no | 276778 | 1459 (0.53%) | 77 [42 - 111] | Reference | Reference | - |
|  | yes | 276778 | 93 (0.03%) | 77 [42 - 111] | 0.06 (0.05 - 0.08) | 0.06 (0.05 - 0.07) | 94% (93% ; 95%) |

Model 0: reference model presented in the manuscript: if one individual of the pair died or had an event of interest, i.e. hospitalisation or in-hospital death, the pair was not censored.

Model 1: model with pair-censoring on death (if one individual of the pair died, the pair was censored).

Model 2: model with pair-censoring on death or event of interest (if one individual of the pair died or had an event of interest, i.e. hospitalisation or in-hospital death, the pair was censored).
