# Supplementary material for: Vaccine effectiveness against severe COVID-19 outcomes within the French overseas territories: A cohort study of 2-doses vaccinated individuals matched to unvaccinated ones followed up until September 2021 and based on the National Health Data System
Source: PLoS One. 2022 Sep 9;17(9):e0274309. doi: 10.1371/journal.pone.0274309 (PMC9462750; doi:10.1371/journal.pone.0274309)
Supplement: S2 Table — Hazard ratios (HRs) were obtained using Cox models taking into account all the variables described in Table 1. (DOCX) [file pone.0274309.s002.docx]

**S2 Table.** Vaccine effectiveness in overseas territories measured as overall reduction of the risk of Covid-19-related hospitalization from day 14 after the 2nd injection for individuals without COVID-19 history. Hazard ratios (HRs) were obtained using Cox models taking into account all the variables described in Table 1.

| **Vaccine exposition** | **Number of subjects** | **Number of event (%)** | **Median follow-up [interquartile range]** | **Adjusted HR** | **% risk reduction** |
| --- | --- | --- | --- | --- | --- |
|  |  |  |  | **(95% CI)** |  |
| no | 264172 | 1444 (0.55%) | 78 [42 - 111] | 1 | - |
| yes | 264172 | 91 (0.03%) | 77 [43 - 112] | 0.06 (0.05 - 0.07) | 94% (93%; 95%) |
