# Supplementary material for: Vaccine effectiveness against severe COVID-19 outcomes within the French overseas territories: A cohort study of 2-doses vaccinated individuals matched to unvaccinated ones followed up until September 2021 and based on the National Health Data System
Source: PLoS One. 2022 Sep 9;17(9):e0274309. doi: 10.1371/journal.pone.0274309 (PMC9462750; doi:10.1371/journal.pone.0274309)
Supplement: S3 Table — (DOCX) [file pone.0274309.s003.docx]

**S3 Table.** Algorithms for identification of chonic conditions.

|  |  |
| --- | --- |
|  | **Main features of pathology identification algorithms** |
| **Lifestyle habits** |  |
| *Smoking* | *Hospitalization or LTD with ICD-10: Z716, F17, T652, Z720 since 2006 or* |
|  | *Reimbursement with ATC N06AX12 or N07BA since 2014 or* |
|  | *Hospitalization with ICD-10: I731, J41, J42, J43, J44, T652 and reimbursement of ATC R03AC18 R03AC19 R03BB04 R03BB05 R03BB06 R03BB07 R03AL04 R03AL05 R03AK04 since 2014 or* |
|  | *Nicotine treatment substitution or reimbursement of a consultation for tobacco addictive treatment since 2006* |
| *Alcoholism* | *Hospitalization or LTD with ICD-10: E244, E512, F10, G312, G621, G721, I426, K292, K70, K860, R780, T51, X45, X65, Y15, Y90, Y91, Y573, Z502, Z714, Z721 in the 5 past years or* |
|  | *Reimbursement ATC N07BB01 N07BB03 N07BB04 N07BB05 since 2014* |
| *Opioid addiction* | *Hospitalization with ICD-10: F11 in the past 5 years or* |
|  | *Reimbursement of specific treatments in the past 5 years* |
| **Comorbidities** |  |
| **Immunosuppressive treatments** |  |
| *Immunosuppressant* | *at least two reimbursements in the past three months before the index date* |
| *Oral corticosteroids* | *at least four reimbursements in the past six months before the index date* |
| **Cardiometabolic** |  |
| *Obesity* | *Hospitalization with ICD-10: E66 (except 'E6603','E6613','E6683','E6693')* |
|  | *Bariatric surgery in the 5 past years: HFCA001, HFCC003, HFFA001, HFFA011, HFFC004, HFFC018, HFGC900, HFKA001, HFKA002, HFKC001, HFMA009, HFMA010, HFMA011, HFMC006, HFMC007, HFMC008, HGCA009, HGCC027 codes (Common Classification of Medical procedures, CCAM in French)* |
| Diabetes | Hospitalization or LTD with ICD-10: E10 to E14 in the past 2 years or |
|  | 3 reimbursements of specific treatments of ATC A10 (except A10BX06) per year, in at least one of the past 2 years or |
|  | Hospitalization with ICD-10: G59.0, G63.2, G73.0, G99.0, H28.0, H36.0, I79.2, L97, M14.2, M14.6, N08.3 in diabetic patients |
| Dyslipidaemia and lipid-lowering treatments | Hospitalization with ICD-10 E78 or at least 3 reimbursements in 2019 of a lipid-lowering treatment, ATC2 C10 |
| Hereditary metabolic diseases or amyloidosis | Hospitalization or LTD with ICD-10: E85 in the past 5 years or LTD in 2019 "Hereditary metabolic diseases" |
| Hypertension | At least 3 reimbursements in 2019 of an antihypertensive treatment |
| Coronary diseases | LTD withICD-10: I20, I21 to I24 in 2019 or hospitalization with ICD-10: I20, I200+0, I21 to I24 in the past 5 years |
| Obliterating arterial disease of the lower limb | Hospitalization with ICD-10: I70.2, I73.9, I74.0, I74.3, I74.4, I74.5 in the past 5 years or LTD with ICD-10: I70, I73, I74 in 2019 |
| Cardiac rhythm or conduction disturbances | Hospitalization with ICD-10: I44, I45, I47 to I49 in the past 5 years or LTD with ICD-10: I44, I45, I47 to I49 in 2019 |
| Heart failure | Hospitalization with ICD-10: I50, I11.0, I13.0, I13.2, I13.9, IK76.1, J81 in the past 5 years or LTD with ICD-10: I50, I11, I13 in 2019 |
| Valvular diseases | Hospitalization with ICD-10: I05 to I08, I34 to I39 in the past 5 years or LTD with ICD-10: I05 to I08, I34 to I39 in 2019 |
| Stroke | Hospitalization with ICD-10: I60 to I64, I67 to I69 in the past 5 years or LTD with ICD-10: I60 to I64, I67 to I69 in 2019 |
| **Respiratory** |  |
| Chronic respiratory diseases (excluding cystic fibrosis) | Hospitalization with ICD-10: J40 to J47, J96 (except J96.0 or J96.9), J98 in the past 5 years or LTD with ICD-10: J40 to J47, J96, J98 in 2019 or at least 3 reimbursements of ATC R03 in 2019 |
| Cystic fibrosis | Hospitalization with ICD-10: E84 in the past 5 years or LTD with ICD-10: E84 in 2019 |
| Haemophilia or severe haemostasis disorders | Hospitalization with ICD-10: D66, D67 in the past 5 years or LTD with ICD-10: D66 to D69 in 2019 |
| Pulmonary embolism | Hospitalization with ICD-10: I26 in the past 5 years |
| **Cancer** |  |
| Female breast cancer (active) | Hospitalization with ICD-10: C50, D01 in the past 2 years or LTD with ICD-10: C50, D01 starting in 2019 or 2018. |
| Female breast cancer (under surveillance) | Hospitalization with ICD-10: C50, D01 in the past 5 years or LTD with ICD-10: C50, D01 starting before 2018. Patients not hospitalized for active breast cancer in 2018 or 2019. |
| Colorectal cancer (active) | Hospitalization with ICD-10: C18 to C20, D01.0, D01.1, D01.2 in the past 2 years or LTD with ICD-10: C18 to C20 starting in 2019 or 2018. |
| Colorectal cancer (under surveillance) | Hospitalization with ICD-10: C18 to C20, D01.0, D01.1, D01.2 in the past 5 years or LTD with ICD-10: C18 to C20 starting before 2018. Patients not hospitalized for active colorectal cancer in 2018 or 2019. |
| Lung cancer (active) | Hospitalization with ICD-10: C33, C34, D02.1, D02.2 in the past 2 years or LTD with ICD-10: C33, C34 starting in 2019 or 2018. |
| Lung cancer (under surveillance) | Hospitalization with ICD-10: C33, C34, D02.1, D02.2 in the past 5 years or LTD with ICD-10: C33, C34 starting before 2018. Patients not hospitalized for active lung cancer in 2018 or 2019. |
| Prostate cancer (active) | Hospitalization with ICD-10: C61, D07.5 in the past 2 years or LTD with ICD-10: C61 starting in 2019 or 2018 or 3 reimbursements of hormonotherapy in 2019 or 2018 (ATC G03HA01, L01CD04, L01XX11, L02AA01, L02AA04, L02AE01, L02AE02, L02AE03, L02AE04, L02AE05, L02BB01, L02BB02, L02BB03, L02BB04, L02BX02, L02BX03, V10BX01, V10XX). |
| Prostate cancer (under surveillance) | Hospitalization with ICD-10: C61, D07.5 in the past 5 years or LTD with ICD-10: C61 starting before 2018. Patients not hospitalized for active prostate cancer in 2018 or 2019. |
| Other cancers (active) | Hospitalization with ICD-10 codes beginning by "C" (except C50 with female sex, C18, C19, C20, C61, C33, C34) or D00 - D09 (except D05 with female sex, D01.0, D01.1, D01.2, D07.5, D02.1, D02.2) in the past 2 years or LTD with ICD-10 codes beginning by "C" (except C50 with female sex, C18, C19, C20, C61, C33, C34) or D00 - D09 (except D05 with female sex) starting in 2019 or 2018. |
| Other cancers (under surveillance) | Hospitalization with ICD-10 codes beginning by "C" (except C50 with female sex, C18, C19, C20, C61, C33, C34) or D00 - D09 (except D05 with female sex, D01.0, D01.1, D01.2, D07.5, D02.1, D02.2) in the past 5 years or LTD with ICD-10 codes beginning by "C" (except C50 with female sex, C18, C19, C20, C61, C33, C34) or D00 - D09 (except D05 with female sex) before 2018. |
| **Inflammatory and skin diseases** |  |
| Chronic inflammatory bowel diseases | Hospitalization with ICD-10: K50, K51, M07.4, M07.5 in the past 5 years or LTD with ICD-10: K50, K51, M07.4, M07.5 in 2019 |
| Rheumatoid arthritis and related diseases | Hospitalization with ICD-10: M06, M06, M08 (except M08.1), M09 in the past 5 years or LTD with ICD-10: M06, M06, M08 (except M08.1), M09 in 2019 |
| Ankylosing spondylitis and related diseases | Hospitalization with ICD-10: M07 (except M07.4, M07.5), M08.1, M45, M46 in the past 5 years or LTD with ICD-10: M07 (except M07.4, M07.5), M08.1, M45, M46 in 2019 |
| *Psoriasis* | *2 reimbursements in the past 2 years of specific treatments (D05AX02, D05AX03, D05AX04, D05AX05, D05AX52)* |
| **Psychological and neurodegenerative diseases** |  |
| Neurotic and Mood Disorders, use of antidepressant treatments | Hospitalization with ICD-10: F30 to F34, F38 to F45, F48 in the past 2 years or LTD with ICD-10: F30 to F34, F38 to F45, F48 in 2019 or 3 reimbursements of antidepressant ATC N06A (except CIP13 3400933338022), Lithium ATC code N05AN01, Dépakote® CIP13 3400934876233, 3400934876691, 340093544427 or Dépamide® ATC code N03AG02 in 2019 |
| Psychotics disorders, use of neuroleptics treatments | Hospitalization with ICD-10: F20 to F25, F28, F29 in the past 2 years or LTD with ICD-10: F20 to F25, F28, F29 in 2019 or 3 reimbursements of ATC N05A (except N05AN01, N05AL06, CIP13 3400932896332) in 2019. |
| Psychiatric disorders starting in childhood | Hospitalization with ICD-10: F80 to F84, F88 to F95, F98 in the past 2 years or LTD with ICD-10: F80 to F84, F88 to F95, F98 in 2019. |
| *Down syndrome* | *Hospitalization or LTD with Q90 in the past 5 years.* |
| Epilepsy | Hospitalization with ICD-10: G40, G41 in the past 5 years or LTD with ICD-10: G40, G41 in 2019 |
| Multiple sclerosis | Hospitalization with ICD-10: G35 in the past 5 years or LTD with ICD-10: G35 in 2019 or 3 reimbursements of specific treatments in 2019 (L03AB07, L03AB08, L03AB13, L03AX13, L04AA23, L04AA27, L04AA31, N07XX07, N07XX09) |
| Paraplegia | Hospitalization with ICD-10: G82 in the past 5 years or LTD with ICD-10: G82 in 2019 |
| Myopathy or myasthenia gravis | Hospitalization with ICD-10: G70 to G73 in the past 5 years or LTD with ICD-10: G70 to G7 in 2019 |
| Parkinson disease | Hospitalization with ICD-10: G20, F02.3 in the past 5 years or LTD with ICD-10: G20 in 2019 or 3 reimbursements of specific treatments in 2019 (CIP13 3400933284398, ATC codes N04BA02, N04BA03, N04BC01, N04BC02, N04BC04, N04BC07, N04BD01, N04BD02, N04BX01, N04BX02 - except CIP7 3644494, 3644502, 3644525, 3644548, 3914841, 3918394, 3918402, 3918425, 3918448, 3927163, 3927217, 3927269, 3927298, 3933442, 3933502, 3933583, 3933637, 3935079, 3935263, 3935346, 3005943). |
| Dementias (including Alzheimer's disease) | Hospitalization with ICD-10: F00 to F03 (except F02.3, F02.4), G30, F05.1 in the past 5 years or LTD with ICD-10: F00 to F03 (except F02.3, F02.4) in 2019 or 3 reimbursements of specific treatments in 2019 or in 2018 (ATC codes N06DA, N06DX01). |
| Mental impairment | Hospitalization with ICD-10: F70 to F73,F78, F79 in the past 2 years or LTD with ICD-10: F70 to F73,F78, F79 in 2019. |
| **Other pathologies** |  |
| HIV infection | Hospitalization with ICD-10: B20 to B24,F02.4, Z21 in the past 5 years or LTD with ICD-10: B20 to B24,F02.4, Z21 in 2019 or 3 reimbursements of specific treatments in 2019 (ATC codes J05AF01, J05AF02, J05AF03, J05AF04, J05AF06, J05AF13, J05AG01, J05AG03, J05AG04, J05AG05, J05AR01, J05AR02, J05AR04, J05AE01, J05AE02, J05AE03, J05AE04, J05AE05, J05AE07, J05AE08, J05AE09, J05AE10, J05AR10, J05AR06, J05AR08, J05AR09, J05AR13, J05AR18, J05AR19, J05AR20, J05AR21, J05AX07, J05AX08, J05AX09, J05AX12) or reimbursement of a specific act of biology medical in 2019 (0805, 0806, 1691, 4117, 4122) |
| Liver diseases | Hospitalization with ICD-10: B18, I85, K70 to F76, Z94.4 in the past 5 years or LTD with ICD-10: B18, I85, K70 to F76, Z94.4 in 2019 or 3 reimbursements of specific treatment of chronic B hepatitis in 2019 (ATC codes J05AF08, J05AF10, J05AF11, UCD codes 9212525, 9212531, CIP7 codes 3519671, 3519694 ) or 1 reimbursement at the same date of interferon and ribavirin in 2019 or 1 delivery of a direct-acting antiviral (DAA) against hepatitis C virus (HCV) in 2019 or 1 reimbursement for HCV genotyping in 2019 or 3 reimbursements for a quantitative HCV RNA PCR, or 2 reimbursements and at least one assessment of hepatic fibrosis. |
| Chronic dialysis | 45 days on hemodialysis or at least 1 day on peritoneal dialysis in 2019 or 1 day of hemodialysis (and less than 45 days) in 2019 AND a treatment duration of at least 45 days of hemodialysis or at least 1 day of peritoneal dialysis during 2018 and without kidney transplant or kidney transplant follow-up in 2019. |
| Renal transplant | Kidney transplant hospitalization regardless of severity level in 2019 or act of kidney transplantation or pancreas and kidney, by laparotomy in 2019 or 3 reimbursements of immunosuppressive drug used for kidney transplant rejection in 2019 or hospitalization for kidney transplantation or monitoring in the past 5 years. |
| *Cardiac transplant* | *Act of cardiac transplantation (DZEA001-DZEA004) in the past 5 years (Common Classification of Medical procedures, CCAM in French)* |
| *Liver transplant* | *Act of liver transplantation (HLEA001, HLEA002) in the past 5 years (Common Classification of Medical procedures, CCAM in French)* |
| *Lung transplant* | *Act of lung transplantation (GFEA001 - GFEA007) in the past 5 years (Common Classification of Medical procedures, CCAM in French)* |
