# Supplementary material for: Vaccine effectiveness against severe COVID-19 outcomes within the French overseas territories: A cohort study of 2-doses vaccinated individuals matched to unvaccinated ones followed up until September 2021 and based on the National Health Data System
Source: PLoS One. 2022 Sep 9;17(9):e0274309. doi: 10.1371/journal.pone.0274309 (PMC9462750; doi:10.1371/journal.pone.0274309)
Supplement: S4 Table — (DOCX) [file pone.0274309.s004.docx]

**S4 Table.** Patients ‘distribution by month of 2^nd^ injection

| **Month of 2nd-injection** | **Number of patients** |
| --- | --- |
| January | 99 |
| February | 4,853 |
| March | 12,249 |
| April | 20,630 |
| May | 41,248 |
| June | 67,417 |
| July | 57,605 |
| August | 72,674 |
| September | 3 |
