# Supplementary material for: Vaccine effectiveness against severe COVID-19 outcomes within the French overseas territories: A cohort study of 2-doses vaccinated individuals matched to unvaccinated ones followed up until September 2021 and based on the National Health Data System
Source: PLoS One. 2022 Sep 9;17(9):e0274309. doi: 10.1371/journal.pone.0274309 (PMC9462750; doi:10.1371/journal.pone.0274309)
Supplement: S5 Table — Hazard ratios (HRs) were obtained using Cox models taking into account all the variables described in Table 1. (DOCX) [file pone.0274309.s005.docx]

**S5 Table.** Vaccine effectiveness in overseas territories measured as overall reduction of the risk of Covid-19-related hospitalization from day 14 after the 2^nd^ injection for individuals fully vaccinated with the BNT162b2 vaccine. Hazard ratios (HRs) were obtained using Cox models taking into account all the variables described in Table 1.

| **Vaccine exposition** | **Number of subjects** | **Number of events (%)** | **Median follow-up [interquartile range]** | **Crude HR** | **Adjusted HR** | **% risk reduction** |
| --- | --- | --- | --- | --- | --- | --- |
|  |  |  |  | **(95% CI)** | **(95% CI)** |  |
| no | 270,569 | 1,402 (0.52%) | 77 [42 - 111] | 1 | 1 | - |
| yes | 270,569 | 94 (0.03%) | 78 [42 - 111] | 0.07 (0.05 - 0.08) | 0.06 (0.05 - 0.08) | 94% (92% ; 95%) |
