# Supplementary material for: Vaccine effectiveness against severe COVID-19 outcomes within the French overseas territories: A cohort study of 2-doses vaccinated individuals matched to unvaccinated ones followed up until September 2021 and based on the National Health Data System
Source: PLoS One. 2022 Sep 9;17(9):e0274309. doi: 10.1371/journal.pone.0274309 (PMC9462750; doi:10.1371/journal.pone.0274309)
Supplement: S6 Table — Hazard ratios (HRs) were obtained using Cox models taking into account age (<75 vs ≥75), gender, combining alcoholism and smoking, frailty and number of comorbidity. (DOCX) [file pone.0274309.s006.docx]

**S6 Table.** Vaccine effectiveness in overseas territories measured as overall reduction of the risk of Covid-19-related and in-hospital death from day 14 after the 2^nd^ injection. Hazard ratios (HRs) were obtained using Cox models taking into account age (<75 vs ≥75), gender, combining alcoholism and smoking, frailty and number of comorbidity.

| **Vaccine exposition** | **Number of subjects** | **Number of event (%)** | **Median follow-up [interquartile range]** | **Adjusted HR** | **% risk reduction** |
| --- | --- | --- | --- | --- | --- |
|  |  |  |  | **(95% CI)** |  |
| no | 276778 | 285 (0.1%) | 77 [42 - 111] | 1 | - |
| yes | 276778 | 19 (0.01%) | 77 [42 - 111] | 0.06 (0.04 - 0.10) | 94% (90%; 96%) |
