# Supplementary material for: Vaccine effectiveness against severe COVID-19 outcomes within the French overseas territories: A cohort study of 2-doses vaccinated individuals matched to unvaccinated ones followed up until September 2021 and based on the National Health Data System
Source: PLoS One. 2022 Sep 9;17(9):e0274309. doi: 10.1371/journal.pone.0274309 (PMC9462750; doi:10.1371/journal.pone.0274309)
Supplement: S1 File — (DOCX) [file pone.0274309.s007.docx]

**Supporting information**

**eMethods**

*Data sources: the French National Health Data System SNDS*

This study was based on the National Health Data System (SNDS), which covers the entire French population, i.e., 67 million inhabitants, and which has been extensively used in France to conduct pharmaco-epidemiology studies, including studies on the COVID-19 pandemic (References below).

Since 2006, a unique, anonymous individual identifier links data derived from two main databases: the DCIR (Datamart de Consommation Inter-Regimes, the national health insurance reimbursement database) and the PMSI (Programme de Médicalisation des Systèmes d’Information, the national hospital database). Each patient is identified by a unique identifier (encrypted twice in the SNDS) for data collection. The INSEE number (INSEE: Institut National de la Statistique et des Études Économiques - French National Institute for Statistics and Economic Studies) is a combination of 13 figures attributed to each person born in France at birth or at the time of legal immigration of any person residing in France. INSEE is responsible for assigning this registration number at the time of civil status registration. All adults legally residing in France have a national health insurance card ("Carte Vitale"), equipped with an electronic chip. When dispensing a drug, the pharmacist scans the bar code on the drug package (brand name, dose strength, number of tablets) and records the patient's identifier by means of the Carte Vitale. Patients must also present their Carte Vitale whenever they are admitted to hospital, as funding of the patient's hospital stay and treatment outside of hospital is dependent on the patient's identifier.

The DCIR includes individual data concerning reimbursements of outpatient medical care, laboratory tests and drugs, coded according to the Anatomical Therapeutic Chemical (ATC) classification. Health expenditures for people with long-term diseases (ALDs), such as cancer or diabetes, is fully covered financially and their diagnoses are registered according to the International Classification of Diseases, 10th Revision (ICD-10).

The PMSI indicates the dates of admission and discharge for all public or private hospital stays in France. Medical diagnoses are coded according to the ICD-10 classification and the main medical or surgical procedures are coded according to the Classification Commune des Actes Médicaux (CCAM).

Patients receiving at least one healthcare reimbursement after January 1, 2018, were identified from the SNDS and were considered to be included in this study. Twins and foreign residents were excluded due to identification difficulties and we also excluded people with missing data for age and/or sex or who died before December 20, 2020.

References of some publications based on data from the SNDS

1. Bouillon K, Bertrand M, Bader G, Lucot J-P, Dray-Spira R, Zureik M. Association of Hysteroscopic vs Laparoscopic Sterilization With Procedural, Gynecological, and Medical Outcomes. JAMA. 2018;319(4):375-387. doi:10.1001/jama.2017.21269
2. Weill A, Dalichampt M, Raguideau F, Ricordeau P, Blotière P-O, Rudant J, Alla F, Zureik M. Low dose oestrogen combined oral contraception and risk of pulmonary embolism, stroke, and myocardial infarction in five million French women: cohort study. BMJ. 2016;353:i2002. doi:10.1136/bmj.i2002
3. Maura G, Blotière P-O, Bouillon K, Billionnet C, Ricordeau P, Alla F, Zureik M. Comparison of the short-term risk of bleeding and arterial thromboembolic events in nonvalvular atrial fibrillation patients newly treated with dabigatran or rivaroxaban versus vitamin K antagonists: a French nationwide propensity-matched cohort study. Circulation. 2015;132(13):1252-1260. doi:10.1161/CIRCULATIONAHA.115.015710
4. Meyer A, Rudant J, Drouin J, Weill A, Carbonnel F, Coste J. Effectiveness and Safety of Reference Infliximab and Biosimilar in Crohn Disease: A French Equivalence Study. Ann Intern Med. 2019;170(2):99-107. doi:10.7326/M18-1512
5. Tubiana S, Blotière P-O, Hoen B, Lesclous P, Millot S, Rudant J, Weill A, Coste J, Alla F, Duval X. Dental procedures, antibiotic prophylaxis, and endocarditis among people with prosthetic heart valves: nationwide population-based cohort and a case crossover study. BMJ. 2017;358:j3776.

*Description of the matching and adjustment variables*

We considered the patient's year of birth, gender, and overseas territories as matching variables. In adjusted Cox proportional hazards models, age was defined as a categorical variable by five-year-age groups. We used the social deprivation index as a measure of socioeconomic status. This indicator is based on the median household income, the percentage of high school graduates in the population over the age of 15, the percentage of manual workers in the labor force, and the unemployment rate for the person's town of residence.

The mapping of diseases and expenditures is a tool developed from the DCIR and PMSI databases, allowing the identification of diseases by means of medical algorithms (47 diseases were studied in the present study) based on the reasons for hospitalization, ALD diagnoses, and/or the reimbursement of specific treatments for certain diseases over the previous four years. The detailed definition of these disease identification algorithms is publicly available in French (<https://www.ameli.fr/fileadmin/user_upload/documents/Methodologie_medicale_cartographie.pdf>). Mapping algorithms allowed the identification of patients presenting a number of distinct diseases in 2020 and were completed by the identification of obese patients, smokers, and people with alcohol-use disorder.

The following 23 chronic diseases were considered in adjusted Cox models: cardiometabolic diseases, such as diabetes, hypertension, dyslipidemia and/or lipid-lowering drug treatment or cardiovascular diseases (stroke and stroke sequelae, heart failure, coronary heart disease, cardiac arrhythmias or conduction disorders, valvular heart disease and peripheral artery disease), chronic respiratory diseases (excluding cystic fibrosis), pulmonary embolism, active cancers (female breast, lung, prostate, colorectal and other cancers), inflammatory diseases (chronic inflammatory bowel disease [IBD], rheumatoid arthritis, ankylosing spondylitis and related diseases), mental and behavioral disorders, neurodegenerative diseases, liver diseases, severe chronic kidney diseases and renal transplant. In the calculation of the number of comorbidities, we further distinguished breast, colorectal, lung, prostate and other active cancers from passive cancers, psoriasis, Down syndrome, multiple sclerosis, paraplegia, myopathy or myasthenia gravis, mental impairment, haemophilia or severe haemostasis disorders, HIV infections, cardiac, liver, lung transplants, i.e a total of 47 comorbidities.

**References**:

Rachas A, Gastaldi-Menager C, Denis P, Lesuffleur T, Nicolas M, Pestel L, Mette C, Drouin J, Riviere S, Tajahmady A, Gissot C, Fagot-Campagna A. Prevalences and healthcare expenditures related to 58 health conditions from 2012 to 2017 in France: diseases and healthcare expenditure mapping, a national population-based study. medRxiv. Published online January 1, 2020:2020.09.21.20198853. doi:10.1101/2020.09.21.20198853

Semenzato L, Botton J, Drouin J, Cuenot F, Dray-Spira R, Weill A, Zureik M. Chronic diseases, health conditions and risk of COVID-19-related hospitalisation and in-hospital mortality during the first wave of the epidemic in France: a cohort study of 66 million people. Lancet Reg Health Eur. 2021 Sep;8:100158. doi: 10.1016/j.lanepe.2021.100158
