# Supplementary material for: Vaccine effectiveness against severe COVID-19 outcomes within the French overseas territories: A cohort study of 2-doses vaccinated individuals matched to unvaccinated ones followed up until September 2021 and based on the National Health Data System
Source: PLoS One. 2022 Sep 9;17(9):e0274309. doi: 10.1371/journal.pone.0274309 (PMC9462750; doi:10.1371/journal.pone.0274309)
Supplement: Attachment — Submitted filename: responses_to_reviewers_v10062022.docx [file pone.0274309.s008.docx]

Dear Editor,

# We would like to thank you and the reviewers for their thorough review which helps to improve our manuscript. We now submit a revised version of our article entitled “Vaccine effectiveness against severe COVID-19 outcomes within the French overseas territories: a cohort study of 2-doses matched vaccinated individuals until September 2021 based on the National Health Data System”. You will find below the point-by-point response to the questions raised by the reviewers.

Reviewer #1: This manuscript presents a matched cohort study based on data from the French National Health Data System. The main finding is that two Covid-19 vaccinations have been highly effective against severe Covid-19 outcomes in 2021 in the French overseas territories. This is important to note as the vaccination coverage in these regions was surprisingly low. Region-specific might help (might already have helped) raising the vaccine uptake and benefiting public health.

We thank the reviewer for the assessment of our manuscript and highlighting the importance of this work.

Major comments:
- The length of the study (median follow-up 77 days) perfectly suits the purpose of demonstrating the benefit of vaccination in 2021 in order to raise vaccination coverage levels. However, since then new variants have emerged, and additional vaccine doses have been broadly recommended. I therefore suggest giving a reference to either the study period or the studied vaccine schedule (2 doses) and length of follow-up in the title.

We have enriched the title as follows: “Vaccine effectiveness against severe COVID-19 outcomes within the French overseas territories: a cohort study of 2-doses matched vaccinated individuals until September 2021 based on the National Health Data System”.

- It is unclear how the matched pair was treated if the unvaccinated individual received a vaccination during follow-up. This needs to be explained.

We agree that this important information was missing in the manuscript for proper understanding.

We added this explanation on page 4 line 23.

‘If a control subject was vaccinated during follow-up, follow-up was stopped for the pair, and the subject was then eligible to be included as a vaccinated subject.’

- The sensitivity analysis which focused on the delta period should be described better. 1. Mention it in the methods section. 2. I assume that it was not only the time period for case/event recruitment that was restricted to July – December but it was the whole study period/follow-up that was restricted. This should be stated correctly and clearly in the abstract (lines 19-20).

In this sensitivity analysis, we considered all pairs without any hospitalisation before July 15, 2021 and focused on events occurring on the delta period, e.g. from July 15, 2021 to September, 30 2021.

In the abstract on page 2 line 23 we specified:

“The results were similar when looking specifically at hospitalizations between July 15 and September 30, 2021”

In the Material and Methods section, we added on page 5 line 7:

“We performed a sensitivity analysis focusing on events occurring over a period where the delta variant was predominant, from July 15 2021, to September 30, 2021, excluding pairs with at least one individual hospitalized before July 15, 2021.”

- Results, lines 17-18: This is unclear. The number of deaths in the text are smaller than any count given in Table 4.

We were referring in this sentence two 2 distinct analyses presented separately in two tables, which was indeed not clear.

We now specify in the revised version of the manuscript on page 5 line 27 that information concerning the number of deaths was presented in Table 2 while the results limited to the period where the delta variant was predominant are presented in Table 3.

- Table 2: % risk reduction is the effect measure that is in the main text referred to as vaccine effectiveness. Please clearly define VE as % risk reduction in the methods and then also mention the term vaccine effectiveness here in the table. Why does the interquartile range of the median follow-up differ between the vaccinated and unvaccinated. Is it because if one individuals of a matched pair died, the other individual was not censored? If so, this might lead to bias. Please state how the variables in Table 1 were taken into account.

We specified in the Material and Methods section on page 4 line 27:

“We estimated vaccine effectiveness after a full vaccination schedule, defined as ≥14 days after the second dose, as the percentage of risk reduction (1-Hazard Ratio) in COVID-19-related hospitalization and in-hospital COVID-19-related death using Cox proportional hazards models adjusted for baseline characteristics and comorbidities.”

Regarding the second point raised, indeed, if one individual of a matched pair died or had an event of interest, the other individual was not censored, which explains slight differences in interquartile range between vaccinated and unvaccinated individuals. We performed additional pair-censoring analyses on death or event of at least one individual in the matched pair. Similar results were obtained and added in the supplementary materials in Table S1.

Table S1: Vaccine effectiveness against COVID-19-related hospitalisation depending on censoring variable.

| **Model** | **Vaccine exposition** | **Number of subjects** | **Number of event (%)** | **Median follow-up [interquartile range]** | **Crude HR** | **Adjusted HR** | **% risk reduction** |
| --- | --- | --- | --- | --- | --- | --- | --- |
|  |  |  |  |  | **(95% CI)** | **(95% CI)** |  |
| 0 | no | 276778 | 1465 (0.53%) | 77 [42 - 111] | Reference | Reference | - |
|  | yes | 276778 | 96 (0.03%) | 77 [42 - 111] | 0.06 (0.05 - 0.08) | 0.06 (0.05 - 0.07) | 94% (93% ; 95%) |
| 1 | no | 276778 | 1459 (0.53%) | 77 [42 - 111] | Reference | Reference | - |
|  | yes | 276778 | 95 (0.03%) | 77 [42 - 111] | 0.06 (0.05 - 0.08) | 0.06 (0.05 - 0.07) | 94% (93% ; 95%) |
| 2 | no | 276778 | 1459 (0.53%) | 77 [42 - 111] | Reference | Reference | - |
|  | yes | 276778 | 93 (0.03%) | 77 [42 - 111] | 0.06 (0.05 - 0.08) | 0.06 (0.05 - 0.07) | 94% (93% ; 95%) |

Model 0: reference model presented in the manuscript: if one individual of the pair died or had an event of interest, i.e. hospitalisation or in-hospital death, the pair was not censored.

Model 1: model with pair-censoring on death (if one individual of the pair died, the pair was censored).

Model 2: model with pair-censoring on death or event of interest (if one individual of the pair died or had an event of interest, i.e. hospitalisation or in-hospital death, the pair was censored).

We added on page 5 line 4:

‘If one individual of a matched pair died or had an event of interest, the other individual was not censored. Additional pair-censoring analyses on death or event of at least one individual in the matched pair were performed in supplementary Table S1.’

How the variables in Table 1 were taken into account is explained in the answer to the next question.

- There are 3 pages of covariates and other than that some of them were used for matching, it is unclear how the others were used. In the methods covariate adjustment is mentioned. But were all the distinct comorbidities considered separately or were they somehow combined? I doubt the study size and case numbers were big enough to allow for that many covariates.

Each individual receiving a first dose of vaccine was matched for year of birth, sex, and overseas territories to one unvaccinated control, randomly selected on the date of vaccination. Then we adjusted simultaneously for the 7 sociodemographic characteristics, history of COVID-19 and the 23 comorbidities presented in the Table 1. We can notice in Table 2 the stability of the models with few differences between crude associations and fully adjusted models.

We specified in the Methods section on page 4 line 27:

“We estimated vaccine effectiveness after a full vaccination schedule, defined as ≥14 days after the second dose, as the percentage of risk reduction (1-Hazard Ratio) in COVID-19-related hospitalization and in-hospital COVID-19-related death using Cox proportional hazards models adjusted for the 7 sociodemographic characteristics, history of COVID-19 and the 23 comorbidities presented in the Table 1”

- Table 3: Is there something missing, is the table legend wrong or were the outcomes hospitalization and mortality combined in this analysis? The outline of the results suggest that you run two models, one for the vaccinated and one for the unvaccinated. Is that correct? In this case you allowed the effect of age and sex to be different in the two groups. Why? Such an effect is not considered in the main VE analysis, is it? It would be great to add the stratified VE estimates, i.e. VE in the group without comorbidities, VE in the group with exactly 1 comorbidity etc. Like this you could demonstrate the benefit of vaccination depending on the number of comorbidities.

We indeed performed two models, one for the vaccinated and one for the unvaccinated, to estimate reduction risk of hospitalisations according to the number of comorbidities.

As another way to examine the impact of the interaction between the number of comorbidities and the vaccination on COVID-19-related hospitalisation, we performed additional analyses of vaccine effectiveness stratified according to the number of comorbidities. Results are presented below and added in Table 5.

We can notice that vaccine effectiveness against COVID-19-related hospitalisations decreases with increasing number of comorbidity from 97% (95% ; 98%) for patients without any comorbidity to 83% (73% ; 89%) for patients with at least 5 comorbidities.

We added a reference to the Table 5 on page 5 line 27:

‘The risk of COVID-19-related hospitalization increased with the number of comorbidities, especially among vaccinated individuals (compared to patients without comorbidity, aHR 29.5 95%CI [13.4-64.9] among vaccinated with 5+ comorbidities and aHR 4.4 95%CI [3.5-5.6] among unvaccinated individuals with 5+ comorbidities) (Table 4). Vaccines were all the more effective among individuals with no or few comorbidities (VE of 97% [95%; 98%] among individuals with no comorbidity vs 83% [73%; 89%] among 5+) (Table 5).’

Table 5: Vaccine effectiveness against COVID-19-related hospitalisation by number of comorbidity.

| **Number of comorbidities** | **Vaccine exposition** | **Number of subjects** | **Number of event (%)** | **Median follow-up [interquartile range]** | **Crude HR** | **Adjusted HR** | **% risk reduction** |
| --- | --- | --- | --- | --- | --- | --- | --- |
|  |  |  |  |  | **(95% CI)** | **(95% CI)** |  |
| 0 | no | 181083 | 458 (0.25%) | 70 [40 - 102] | 1 | 1 | - |
|  | yes | 168704 | 12 (0.01%) | 68 [38 - 99] | 0.03 (0.02 - 0.05) | 0.03 (0.02 - 0.05) | 97% (95% ; 98%) |
| 1 | no | 46258 | 309 (0.67%) | 85 [48 - 118] | 1 | 1 | - |
|  | yes | 50546 | 11 (0.02%) | 85 [48 - 119] | 0.03 (0.02 - 0.06) | 0.03 (0.02 - 0.06) | 97% (94% ; 98%) |
| 2 | no | 23202 | 261 (1.12%) | 91 [54 - 125] | 1 | 1 | - |
|  | yes | 26411 | 18 (0.07%) | 94 [57 - 127] | 0.06 (0.04 - 0.10) | 0.06 (0.04 - 0.10) | 94% (90% ; 96%) |
| 3 | no | 14627 | 224 (1.53%) | 95 [57 - 129] | 1 | 1 | - |
|  | yes | 17447 | 15 (0.09%) | 98 [62 - 132] | 0.06 (0.03 - 0.09) | 0.05 (0.03 - 0.09) | 95% (91% ; 97%) |
| 4 | no | 6818 | 107 (1.57%) | 97 [58 - 132] | 1 | 1 | - |
|  | yes | 8153 | 15 (0.18%) | 101 [66 - 140] | 0.11 (0.06 - 0.19) | 0.10 (0.05 - 0.17) | 90% (83% ; 95%) |
| 5 | no | 4790 | 106 (2.21%) | 99 [61 - 134] | 1 | 1 | - |
|  | yes | 5517 | 25 (0.45%) | 106 [70 - 143] | 0.18 (0.12 - 0.28) | 0.17 (0.11 - 0.27) | 83% (73% ; 89%) |

- Discussion, line 22: This has not been shown. You need to show the VE stratified by the number of comorbidities, or you rephrase the second part of the sentence.

We added a reference to Table 5 describing VE stratified by the number of comorbidities on page 7 line 3:

‘Vaccines were highly effective, but even more so among those with no or few comorbidities (Table 5)’

Minor comments:
- The outcome or events of interest are hospitalization or death. The effect measure is vaccine effectiveness against these outcomes (not against the risk). Please correct this in the abstract and elsewhere, where you write a) that the outcome was vaccine effectiveness and b) that you estimated vaccine effectiveness against the risk of hospitalization/death. The table captions where you refer to the reduction of the risk are perfectly fine though.

We corrected in the Method section of the Abstract the sentence as follows:

‘Events of interest were COVID-19-related hospitalization and in-hospital death. We then estimated vaccine effectiveness against COVID-19-related hospitalization after a full vaccination schedule, defined as ≥14 days after the second dose, and against in-hospital COVID-19-related death.’

Several ‘against the risk of’ were suppressed in the manuscript.

- Please carefully review the terminology and use the terms consistently: e.g., vaccine effectiveness vs. vaccination effectiveness; Guiana vs. Guyana

We corrected in the manuscript the specific terms mentioned.

- Abstract, line 13: Split the sentence into two sentences and clearly state how number of comorbidities was taken into account.

We specified in the Methods section of the Abstract:

‘We then estimated vaccine effectiveness against COVID-19-related hospitalization after a full vaccination schedule, defined as ≥14 days after the second dose, and against in-hospital COVID-19-related death. Finally, we estimated the risk of COVID-19-related hospitalization among vaccinated and unvaccinated individuals respectively, and vaccine effectiveness stratified according to the number of comorbidities, defined as a categorical variable ranging from 0-5 or more, based on the sum of 47 identified chronic conditions.’

- Replace “ more significantly” by other phrases that would express that the effect in vaccinated is stronger

We propose to replace “more significantly” by “especially” on page 5 line 28:

‘The risk of COVID-19-related hospitalization increased with the number of comorbidities, especially among vaccinated individuals.’

- Choose either “Reference” or “1” for the ratio estimates of the reference group

We choose 1 for the ratio estimates of the reference group.

Reviewer #2: Dear Editors of Plos One and authors of manuscript,
Semenzato et al present vaccine effectiveness estimates from French overseas territories. The manuscript is well written and the analyzes is conducted in good manner. However, I would like to point out some improvement:

We thank the reviewer for the evaluation of our manuscript and for the advice.

Major comments:

1) I would like to see in a figure what is the time since the second dose among the vaccinated (for example the N of two dose vaccinated in every month).

You will find below the distribution of patients according to the month of full-vaccination. We added the table in Supplementary material in Table S3

| **Month of 2nd-injection** | **Number of patients** |
| --- | --- |
| January | 99 |
| February | 4,853 |
| March | 12,249 |
| April | 20,630 |
| May | 41,248 |
| June | 67,417 |
| July | 57,605 |
| August | 72,674 |
| September | 3 |

We corrected in the Abstract and the Results section from ‘In total, 276,778 individuals had a double-dose vaccination by July, 31 (Table S3) and were followed with their unvaccinated counterparts.’ to ‘In total, 276,778 vaccinated individuals had a double-dose vaccination during the follow-up period and were followed with their paired unvaccinated control’ and added a reference to the table on page 5 line 14.

2) How is the area of residency defined? Is this municipality based or just the overseas territories? I don’t understand this from

We adjusted analyses for overseas territories; we specified this in the manuscript.

3) Were the individuals with prior SARS-CoV-2 infection to 27th Dec included in the study? They should be excluded or somehow taken into account in the analysis (adjusting or stratification).

Vaccination against COVID-19 started in December, 27^th^ 2020 in France, that’s why individuals with prior infection to this date were not included in the study.

You will find in the Methods section of the Manuscript the restriction of the inclusion period to patients receiving a first vaccine injection ‘between December 27, 2020, and July 31, 2021’.

4) How is the unvaccinated defined? Unvaccinated in the end of follow-up or at the time of matching? This is unclear.

‘For each individual receiving a first dose of COVID-19 vaccine between December 27, 2020, and July 31, 2021, one unvaccinated control matched for year of birth, sex, and overseas territories, was randomly selected on the date of vaccination (first injection). ‘ (on page 4 line 20)

An unvaccinated control was then found at the time of matching.

For more clarification, we added the following sentence on page 4 line 27:

‘If a control subject was vaccinated during follow-up, follow-up was stopped for the pair, and the subject was then eligible to be included as a vaccinated subject.’

5) I would like to see a table of comorbidities used in table 3. If I understood correctly, they are defined in prior article, but it is laborious to check this in prior article.

We added in the Method section on page 5 line 8 a reference to the supplementary Table S2 containing algorithms for the identification of chronic pathologies used in the calculation of the number of comorbidities.

6) What was the time from positive SARS-CoV-2 sample to Covid-19 hospitalization? Were all hospitalized or died persons tested for SARS-CoV-2 infection?

Only 755/1,561 individuals (48%) had a positive SARS-CoV-2 test registered in the SIDEP database before or at the time of hospitalization: 712/1,465 (49%) among unvaccinated individuals and 43/1,465 (45%) among vaccinated individuals. This figure were of 113/285 (40%) and 9/19 (47%) for patients unvaccinated and vaccinated who died at hospital respectively.

For patients with a positive SARS-CoV-2 test registered in the SIDEP database before or at the time of hospitalization, the test was performed on average in the 5 days preceding hospitalisation (interquartile range 2 – 7 days).

We added on page 5 line 24:

‘45% and 40% respectively had a SARS-COV-2 positive test, which occurred on average 5 days before hospitalization.’

7) I don’t understand how the matching for age was done. In the manuscript it is written “2021, one unvaccinated control matched for year of birth” and in supplements “Age was defined as a categorical variable by five-year-age groups”. Was the age matched with each birth year or +/- 5 years from birth?

For each individual receiving a first dose of COVID-19 vaccine between December 27, 2020, and July 31, 2021, one unvaccinated control matched for year of birth, sex, and overseas territories, was randomly selected on the date of vaccination (first injection). In Cox proportional hazards models adjusted for all baseline characteristics and comorbidities, we considered age as a categorical variable by five-year-age groups.

We specified in supplementary material on page 3 line 2:

‘We considered the patient's year of birth, gender, and overseas territories as matching variables. In adjusted Cox proportional hazards models, age was defined as a categorical variable by five-year-age groups’

Minor comments:

1) Results: ”We chose not to exclude the few patients vaccinated with the Ad26.COV2.S vaccine whose vaccination schedule was first in a single injection before requiring a booster dose from late summer.” This is methods.

We moved this sentence in the Method section.

2) In the supplements it is written ” We used the social deprivation index as a measure of socioeconomic status. This indicator is based on the median household income, the percentage of high school graduates in the population over the age of 15, the percentage of manual workers in the labor force, and the unemployment rate for the person's town of residence.” Which part did the authors used this social deprivation index? It is unclear.

Each individual receiving a first dose of vaccine was matched for year of birth, sex, and overseas territories to one unvaccinated control, randomly selected on the date of vaccination. Then we adjusted simultaneously for the 7 sociodemographic characteristics, history of COVID-19 and the 23 comorbidities presented in the Table 1. We can notice in Table 2 the stability of the models with few differences between crude associations and fully adjusted models.

We specified in the Methods section on page 4 line 27:

“We estimated vaccine effectiveness after a full vaccination schedule, defined as ≥14 days after the second dose, as the percentage of risk reduction (1-Hazard Ratio) in COVID-19-related hospitalization and in-hospital COVID-19-related death using Cox proportional hazards models adjusted for the 7 sociodemographic characteristics, history of COVID-19 and the 23 comorbidities presented in the Table 1”

Social deprivation index was one of the several adjustment variables in Cox proportional hazards models.

We renamed the section in the supplements as ‘Description of the matching and adjustment variables‘.

3) Table 3: There is quite significant difference of risk among vaccinated with 5 or more comorbidities compared to unvaccinated as the authors discussed. Have the authors checked if number of comorbidities is higher among vaccinated (if vaccinated had for example 8 comorbidities on average compared 6 among unvaccinated)?

We can already observe in Table 4 that the proportion of patients with much comorbidity is quite similar (slightly higher) between vaccinated and unvaccinated patients: 5% of vaccinated patients had 4 or more comorbidities vs 4% of unvaccinated patients.

The average number of comorbidity was 0.79 among vaccinated patients and 0.69 among unvaccinated patients. When we focused on patients with 5 or more comorbidities, there was no difference on the average number of comorbidities between vaccinated and unvaccinated individuals (5.63 vs 5.65, respectively).
