# Supplementary material for: Vaccine effectiveness against severe COVID-19 outcomes within the French overseas territories: A cohort study of 2-doses vaccinated individuals matched to unvaccinated ones followed up until September 2021 and based on the National Health Data System
Source: PLoS One. 2022 Sep 9;17(9):e0274309. doi: 10.1371/journal.pone.0274309 (PMC9462750; doi:10.1371/journal.pone.0274309)
Supplement: Attachment — Submitted filename: reviewing_v29072022.docx [file pone.0274309.s009.docx]

Dear Editor,

# Again, we would like to thank you and the reviewers for all the comments that allowed improving our manuscript. We now submit a revised version of our article entitled “Vaccine effectiveness against severe COVID-19 outcomes within the French overseas territories: a cohort study of 2-doses vaccinated individuals matched to unvaccinated ones followed up until September 2021 and based on the National Health Data System.” You will find below the point-by-point response to the questions raised by the reviewers.

Reviewer #1: The methods section of the abstract could be slightly shortened: After listing the events of interest, they don’t have to be repeated in the next sentence about vaccine effectiveness. In addition, it’s enough to say that the analysis was stratified by number of comorbidities. No need to give the exact definition of number of comorbidities here in the abstract. As this will lower the word count, please bring back the phrase “the risk of” in the last sentence of the results section and the first two sentences of the conclusions. Also, in the main text “the risk of” should have not been deleted on page 36 lines 9-10, in the very last paragraph (conclusion), and the legends of table 2, 3, and 5. Table 5 is missing the “or more” phrase.

We thank the reviewer for this second evaluation of our manuscript.

We changed the methods section of the abstract as follows:

‘The French National Health Data System was used to conduct a 1:1 matched-cohort study. For each individual receiving a first dose of BNT162b2, mRNA-1273, ChAdOx1 nCoV-19, or Ad26.COV2-S vaccine between December 27, 2020, and July 31, 2021, one unvaccinated individual was randomly selected and matched for year of birth, sex, and overseas territories on the date of vaccination. We estimated vaccine effectiveness against COVID-19-related hospitalization and in-hospital death after a full vaccination schedule, defined as ≥14 days after the second dose. Analyses were also stratified according to the number of comorbidities.’

As suggested, we bring back the phrase ‘the risk of’ in all the sentences/titles mentioned by the reviewer and we added ‘or more’ in Table 5.

I didn’t find an explanation for why this specific set of 23 comorbidities was selected (“considered”) as covariates in the Cox model while the other available comorbidities were only counted in the overall number of comorbidities. The thought behind this selection should be explained. The comorbidities must have been selected a priori and not based on the data.

We did not select comorbidities to include in the models based on the data. As you pointed out, the strategy adopted was different when the comorbidities were adjustment variables than when they were summarized in the variable number of comorbidities. Indeed, for example, Cox models were adjusted on active cancers whereas we distinguished breast, colorectal, lung, prostate and other cancers, active or passive, in the calculation of the number of comorbidities. It enabled us to limit the number of covariates to include in the Cox models, as pointed out in your next question. For the calculation of the number of comorbidities, there was no reason to reduce information *a priori,* and this would even be misleading because the number of comorbidities per person would have been underestimated.

We specified this on page 3 line 17 of the supplementary materials:

‘The following 23 chronic diseases were considered in adjusted Cox models: cardiometabolic diseases, such as diabetes, hypertension, dyslipidaemia and/or lipid-lowering drug treatment or cardiovascular diseases (stroke and stroke sequelae, heart failure, coronary heart disease, cardiac arrhythmias or conduction disorders, valvular heart disease and peripheral artery disease), chronic respiratory diseases (excluding cystic fibrosis), pulmonary embolism, active cancers (female breast, lung, prostate, colorectal and other cancers), inflammatory diseases (chronic inflammatory bowel disease [IBD], rheumatoid arthritis, ankylosing spondylitis and related diseases), mental and behavioural disorders, neurodegenerative diseases, liver diseases, severe chronic kidney diseases and renal transplant. In the calculation of the number of comorbidities, we further distinguished breast, colorectal, lung, prostate and other active cancers from passive cancers, psoriasis, Down syndrome, multiple sclerosis, paraplegia, myopathy or myasthenia gravis, mental impairment, haemophilia or severe haemostasis disorders, HIV infections, cardiac, liver, lung transplants, i.e a total of 47 comorbidities.’

Indeed, covariate adjustment has almost no effect on the estimates. This should be briefly discussed. Is it because matching controlled for most of the differences between vaccinated and unvaccinated? The number of covariates is high. A rule of thumb states that 10-15 events are required per parameter to estimate (or control for) the parameter’s effect. There are alone 13 age groups (12 parameters) and 5 territories (4 parameters) in addition to more than 20 binary covariates (1 parameter each). Due to the high number of hospitalizations among the unvaccinated, the number of hospitalizations seems high enough for this complex model. However, the death counts are much lower. Also, many comorbidities or conditions (e.g. alcoholism) are very rare (<2%) in the cohort. Therefore, I really doubt that cohort size and incidence were high enough for this model. Please either discuss this problem as a potential limitation and/or run another sensitivity analysis (for the supplementary material) in which you don’t adjust for that many covariates by only using the number of comorbidities or using only the frailty indicator and perhaps by combining alcoholism and smoking (because adjusting for highly correlated covariates can negatively affect the variance of the estimates).

This is true that covariate adjustment had almost no effect on the estimates. Since age is a determinant of comorbidities and a strong risk factor of COVID-19, this is not so surprising that we obtained similar estimations between crude and fully adjusted model.

We had a low number of deaths among exposed and unexposed, 19 and 285 respectively. The reviewer is right that incidence is not high enough for most of the comorbidities in adjusted Cox model according to the rule of thumb. However, models seemed not be so unstable, probably because of the number of subjects overall. Nevertheless, we have performed a sensitivity analysis in which we only adjust on age (<75 vs ≥75), gender, combining alcoholism and smoking, frailty and number of comorbidity, and result obtained was similar (94% (90%; 96%) vs 94% (91%; 96%)).

**S6 Table**. Vaccine effectiveness measured as overall reduction of the risk of Covid-19-related and in-hospital death from day 14 after the 2nd injection. Hazard ratios (HRs) were obtained using Cox models taking into account age (<75 vs ≥75), gender, combining alcoholism and smoking, frailty and number of comorbidity.

| **Region** | **Vaccine exposition** | **Number of subjects** | **Number of event (%)** | **Median follow-up [interquartile range]** | **Adjusted HR** | **% risk reduction** |
| --- | --- | --- | --- | --- | --- | --- |
|  |  |  |  |  | **(95% CI)** |  |
| Overseas territories | no | 276778 | 285 (0.1%) | 77 [42 - 111] | 1 | - |
|  | yes | 276778 | 19 (0.01%) | 77 [42 - 111] | 0.06 (0.04 - 0.10) | 94% (90%; 96%) |

We added on page 7 line 4:

‘Vaccine effectiveness against in-hospital death was similar when adjusting for all the variables (Table 2) or when restricting to a few ones (S6 Table): 94% (95%CI [91-96]), with 19 deaths among vaccinated and 285 among unvaccinated individuals; and when restricting the study period from July 15 2021, to September 30, 2021, only (Table 3).’

Last but not least, please briefly discuss the potential flaws of the data. Electronic health records strongly depend on health care seeking behavior and might be incomplete.

We added on page 8 line 28:

‘We may have a misclassification bias due to differential health care seeking behavior between vaccinated individuals and unvaccinated controls. This may lead to a potential underestimation of illness among unvaccinated individuals and then an underestimation of vaccine effectiveness. Health care seeking behavior may also be considered as a confounding factor: people with less use of healthcare would be less vaccinated and hospitalized for covid-19. However, this would have a limited impact due to the severity of the endpoint, and would then lead to an underestimation of vaccine effectiveness. Our study may also have been affected by other residual confounding due to differences between vaccinated individuals and unvaccinated controls after matching and adjustment for a high range of comorbidities that were shown to be associated with COVID-19-related hospitalization.’

Reviewer #2: Dear editors and authors,

We thank the reviewer for this second evaluation of our manuscript.

I thank Semenzato et al for providing the answers for my questions regarding their manuscript. The answers are good and comprehensive. I still have few minor comments:
1) Discussion: COVID-19 related hospitalization and the number of comorbidities should not be interpreted as vaccines failure.
a. I think ”vaccine failures” is correct

We made the proposed change.

2) Tables 2, 3 and 5: Column ”% risk reduction”
a. In my mind it would be better to write ”vaccine effectiveness”
b. I would also name tables 3 and 5 like table 2 to harmonize the writing style◊ ”Vaccine effectiveness measured as overall…”.

We homogenized the titles of the tables as suggested.

3) What is the rationality to use 5 year age as a categorical variable by five-year-age groups in adjusting? Why not continuous variable?
a. However, I think this will not make huge difference for the results.

We choose to use age as a as a categorical variable by five-year-age groups. This choice facilitates the description of the distribution of individuals, while keeping precise information, with age groups sufficiently restricted to avoid residual confusion but sufficiently broad to avoid a lack of subjects.

4) I understand from the answers that you exclude those with prior SARS-CoV-2 infection from the analysis. Is this mentioned in the manuscript?

I’m sorry, I misunderstood your question in the first revision and then gave you an incorrect answer.

We had already taken individuals with previous SARS-CoV-2 infection by adjusting for COVID-19 history in the Cox models (cf Tables 1 and 2), as you suggest in the first revision:

‘Were the individuals with prior SARS-CoV-2 infection to 27th Dec included in the study? They should be excluded or somehow taken into account in the analysis (adjusting or stratification).’

We added here a sensitivity analysis on vaccine effectiveness measured as overall reduction of the risk of Covid-19-related hospitalization by excluding pairs of which at least one individual had a COVID-19 history and obtained similar results.

**S2 Table**: Vaccine effectiveness measured as overall reduction of the risk of Covid-19-related hospitalization from day 14 after the 2nd injection for individuals without COVID-19 history. Hazard ratios (HRs) were obtained using Cox models taking into account all the variables described in Table 1.

| **Region** | **Vaccine exposition** | **Number of subjects** | **Number of event (%)** | **Median follow-up [interquartile range]** | **Adjusted HR** | **% risk reduction** |
| --- | --- | --- | --- | --- | --- | --- |
|  |  |  |  |  | **(95% CI)** |  |
| Overseas territories | no | 264172 | 1444 (0.55%) | 78 [42 - 111] | 1 | - |
|  | yes | 264172 | 91 (0.03%) | 77 [43 - 112] | 0.06 (0.05 - 0.07) | 94% (93%; 95%) |

We added on page 6 line 9:

‘Then, we excluded pairs with at least one individual having COVID-19 history (S2 Table).’

We added on page 7 line 6:

‘Similar results were also obtained when restricting the study period from July 15 2021, to September 30, 2021, only (Table 3) or when excluding pairs with at least one individual having COVID-19 history (S2 Table)’

5) The manuscript doesn’t have strengths and limitations part in the discussion. I would think that the manuscript would benefit from this. I would argue that one of the weakness is that all Covid-19 hospitalized did not have positive SARS-CoV-2 sample in the registers. One strength would be that you have quite comprehensive registers in use.
a. I’m sorry that I did not comment on this in the first revision round

We added the following paragraph in the discussion section:

‘The SNDS is a comprehensive claims database that allowed us to analyse vaccine effectiveness for individuals from the whole population of the French overseas territories benefiting from a 2-dose vaccination schedule, thus limiting selection bias. We focused on patients requiring admission specifically for SARS-CoV-2 infection, and due to the invoicing nature of the codifications recorded by public and private healthcare establishments, diagnostic errors are likely to be rare. Given the predominance of the Delta variant over the period analyzed, our estimates may not apply to periods of high circulation of other variants, such as Omicron. We may have a misclassification bias due to differential health care seeking behavior between vaccinated individuals and unvaccinated controls. This may lead to a potential underestimation of illness among unvaccinated individuals and then an underestimation of vaccine effectiveness. Health care seeking behavior may also be considered as a confounding factor: people with less use of healthcare would be less vaccinated and hospitalized for covid-19. However, this would have a limited impact due to the severity of the endpoint, and would then lead to an underestimation of vaccine effectiveness. Our study may also have been affected by other residual confounding due to differences between vaccinated individuals and unvaccinated controls after matching and adjustment for a high range of comorbidities that were shown to be associated with COVID-19-related hospitalization.’

The fact that not all Covid-19 hospitalized patients had a positive SARS-CoV-2 sample in the registers is not really a limitation. We focused on patients requiring admission specifically for SARS-CoV-2 infection, and due to the invoicing nature of the codifications recorded by public and private healthcare establishments, diagnostic errors are likely to be rare. Codification errors concerning the reason for hospitalization admission would indeed constitute a fraud from the healthcare establishment, which could then suffer heavy financial penalties. The control of these important financial flows by the paying agencies then contributes to the good quality of the coding.
